# Conservative management of upper tract transitional cell carcinoma

**DOI:** 10.4103/0970-1591.40608

**Published:** 2008

**Authors:** Markian R. Iwaszko, Amy E. Krambeck

**Affiliations:** Department of Urology, Mayo Clinic, Rochester, MN, USA

**Keywords:** Carcinoma, endoscopy, transitional cell, ureteral neoplasms, ureteroscopy

## Abstract

**Aim:**

Our aim was to review the current literature describing the endoscopic management of upper tract transitional cell carcinoma (TCC).

**Materials and Methods:**

Review of published, peer-reviewed articles relating the primary ureteroscopic or percutaneous management of upper tract TCC was performed using the MEDLINE database.

**Results:**

Historically, the gold-standard management for upper tract TCC consists of nephroureterectomy with excision of a bladder cuff. The employment of endoscopic management with these neoplasms was initially instituted in individuals with imperative indications, including bilateral disease, solitary kidney, and/or renal insufficiency. For individuals treated with ureteroscopy, recurrence rates range from 30 to 71% and cancer-specific survival rates from 50 to 93%. Results are dependent primarily on tumor grade and stage. In individuals with low-stage, low-grade tumors treated percutaneously, recurrence rates, and cancer-specific survival rates are 18-33% and 94-100%, respectively. Adjuvant therapy has been employed with thiotepa, mitomycin, and BCG, but none have been able to demonstrate a statistically significant difference in recurrence or cancer-specific survival rates.

**Conclusions:**

Endoscopic management is a safe and effective treatment alternative to nephroureterectomy in the management of upper tract TCC. Survival outcomes are comparable, but renal preservation therapy offers the advantage of reduced morbidity, complications, and the potential for better quality of life. Recurrence and disease progression are not uncommon and underscore the need for strict tumor surveillance.

## INTRODUCTION

Upper tract transitional cell carcinoma (TCC) is relatively uncommon, accounting for 5-10% of renal tumors and 5% of all urothelial cancers.[1,2] Upper tract disease increases with age and the mean age at diagnosis is approximately 65 years old.[3] Treatment is dependent on the individual and operative planning is made with consideration to location, grade, and stage of disease. Historically, the gold-standard is nephroureterectomy with excision of a bladder cuff.[4] Unfortunately, in certain instances this will leave the individual anephric and requiring subsequent dialysis. With the goal of renal preservation, the alternative of segmental ureterectomy with primary ureteroneocystostomy may be performed or, if the primary tumor is more proximal, a vesico-psoas hitch or Boari flap may be necessary to obtain more length. If multifocal tumor is present, longer ureteral segments may need to be excised with an ileal ureteral substitution. Alternatively, total ureterectomy with pyelocystostomy or renal autotransplantation to the iliac fossa may be performed.[5] Pathologic review of nephroureterectomy specimens with upper tract TCC demonstrated a large number with low grade and stage disease. These findings provided the impetus to develop more conservative renal preservation techniques for upper tract TCC.[6]

With the development of flexible ureteroscopes, improved optics, and small diameter laser fibers, endoscopic renal preserving treatment is feasible for upper tract TCC. Initially, endoscopic management of ureteral and renal pelvis TCC was reserved for individuals with imperative indications, including bilateral disease, solitary kidney, or renal insufficiency.[7] However, recent studies have demonstrated that endoscopic management may be a safe alternative to nephroureterectomy or segmental ureteral resection in patients with a normal contralateral kidney.[8]

## DIAGNOSIS

Initial presenting symptoms may vary, but hematuria is the most common, either gross or microscopic and is present in 75% of individuals.[1] This is followed by flank pain, which may be present in approximately 30% of individuals at presentation. Many are asymptomatic and others may manifest constitutional symptoms such as malaise, weight loss, and anorexia secondary to advanced or metastatic disease. The diagnosis of upper tract TCC may pose a significant challenge to the physician. Typically, the diagnostic evaluation includes urinalysis, urine cytology, cystoscopy, excretory urography and/or CT urography, and direct endoscopic evaluation with tissue sampling.

The utility of voided urine cytology is limited as it is nonspecific. However, even with the use of selectively obtained ureteral cytologies, the diagnostic yield is still only 60% accurate.[9] Improved diagnostic yield has been demonstrated with the use of saline washing or brush biopsy, with an approximate sensitivity of 90% and specificity near 90%.[10] The concurrent use of imaging modalities allows for the delineation of the pelvicaliceal system and ureters. Historically, excretory urography was the noninvasive method of choice. This diagnostic modality, however, is rapidly being replaced by CT urography. The improved resolution, evaluation of adjacent structures, surveillance of the entire urinary tract, and the ability to capture multiple phases of contrast excretion offer an improved diagnostic potential of CT urography over excretory urography.[11] The ability to detect upper tract malignancies is dependent upon size, as small filling defects (<5 mm) may be missed on the traditional cuts of CT imaging. However, the sensitivity of CT urography in detecting upper tract TCC has been reported to approach 100 with 60% specificity.[12] The typical manifestations of an upper tract tumor are either a radiolucent filling defect, nonvisualization of the collecting system or obstruction of the upper tract. The differential diagnosis may be narrowed by virtue of the varying densities of stones, blood clots, or tumors. However, when the diagnosis remains in question or the treatment plan may be modified based on ureteroscopic evaluation, then endoscopy with or without biopsy should be performed.

Ureteroscopy provides a valuable tool in the evaluation of upper tract TCC. With the advancement in optics, flexible ureteroscopes and endoscopic equipment, visualization, and sampling of the tumor has improved. The greatest prognostic factors in the management of upper tract TCC are pathologic grade and stage. Histologic correlations of up to 90% have been established between the initial ureteroscopic biopsy and the final pathologic specimen.[13] However, due to the small size of the biopsy specimen and depth of tissue sampling, outcomes with tumor stage have not demonstrated such a strong correlation. In 40 urothelial tumors staged in one series, 45% of tumors thought to be pathologic Ta were upstaged to T1 to T3 at the time of complete resection.[14] Ureteroscopic biopsy cannot reliably predict tumor stage. Thus, a combination of tumor grade, endoscopic visual appearance of the tumor, and radiologic appearance are required for the best prediction of tumor stage.

## URETEROSCOPIC MANAGEMENT

Retrograde ureteroscopic management of upper tract UC provides the advantage of reduced morbidity, the ability to perform the procedure on an outpatient basis, and the maintenance of a closed urinary system. The limitations of ureteroscopy, however, are secondary to the instrument size, which allows for a smaller field of view, smaller working channel, and the size of tumor that may be effectively resected. In addition, location of tumor in the lower pole of the renal pelvis is not always reliably accessible and prior urinary diversion may make retrograde access challenging.

There are multiple methods by which upper tract TCC may be treated endoscopically. However, regardless of the method, adequate tissue sampling, and abdominal imaging should be obtained first to rule out the presence of grade 3 and/or T2 disease, both of which are contraindications to endoscopic management. Ureteroscopic biopsies may be performed using a variety of endoscopic instruments.[15] Tumor biopsies may be obtained using 3 French cup biopsy forceps, a flat-wire basket, 3 French snare, grasper, brush, or aspiration catheter, depending on the architecture of the lesion. For example, a flat wire basket or a 3 French snare may provide good sampling for a papillary ureteral tumor whereas a 3 French cup biopsy forceps or brush may be better suited for biopsying a sessile or flat lesion. We recommend brushing the tumor in addition to ureteroscopic biopsy. This allows for cytologic grading determination even if the biopsy tissue sample is too small for adequate pathologic evaluation.

The principles of treatment are similar to those for endoscopic resection of bladder tumors. Effective tissue sampling should be obtained for pathologic diagnosis and subsequent ablation of residual tumor should then be performed. Tumor resection to its base may be accomplished with an ureteroscopic resectoscope in the distal ureter. This differs from bladder tumor resection in that only intraluminal tumor is removed and no attempt is made to obtain deep tissue. Proximal lesions are less amenable to this technique due to the design of the rigid ureteroscope. Alternatively, the lesion may be treated with electrocautery. The use of high cutting current may be used when the lesion is nearly circumferential to reduce the development of scar tissue and subsequent stricture formation. More recently, upper tract lesions in the ureter and renal pelvis have been treated with the neodymium:yttrium-aluminum-garnet (Nd:YAG) and the holmium:YAG (Ho:YAG) lasers.[16] A combination of these two energy sources may be employed to ablate the tumor. Some advocate the use of the Nd:YAG laser initially, due to its greater depth of penetration (4-6 mm), to coagulate the tumor and then utilize the Ho:YAG, with less tissue penetration (<0.5 mm), to allow for more focused ablation.[17]

The endoscopic approach to managing upper tract TCC was first employed in patients with imperative indications, including bilateral disease, solitary kidney, or renal insufficiency with creatinine >2.0 mg/dl.[7] Upper tract and bladder recurrence rates for patients treated endoscopically have been reported to be 30-40% and 35-40%, respectively, regardless of upper tract tumor location.[18,19] Additional studies with limited follow-up have demonstrated an 86-93% survival rate for patients with upper tract TCC treated endoscopically.[20,21] A recent study from our institution, however, has demonstrated that cancer-specific survival in these patients is approximately 50% at 5 years; recurrent disease is also common, with 5-year local recurrence-free survival of 27% and bladder recurrence-free survival of 54%.[22] The utilization of nephroureterectomy in this patient population is an alternative but would render the patient functionally anephric with the requirement of subsequent dialysis. Although small case series describe short-term progression-free survival,[23] dialysis is not without complications and may be associated with a poor quality of life. Endoscopic management offers the benefit of reduced morbidity, complications and the potential for a better quality of life in this patient population.

Due to enhanced technology and increased experience, the role of endoscopic management has become increasingly utilized, even in the presence of a normal contralateral kidney.[8,24] For individuals who have upper tract disease treated electively, cancer-specific and recurrence-free survival rates are more promising. Deligne and associates demonstrated a local recurrence-free survival of 68% and an overall cancer-specific survival of 84%.[25] This is comparable to data published in other series.[20,26,27] However, a more recent large cohort study indicates a higher rate of recurrence: at 5 years, local recurrence-free survival is <40%, bladder recurrence-free survival is 50%, and cancer-specific survival is comparable at 85% (Thompson, unpublished data). The discrepancy in local recurrence-free survival in this study is likely secondary to a longer duration of follow-up. Despite the frequency of recurrence identified in the current literature, in properly selected patients, renal preservation rates are high, reaching approximately 80%.[20,25]

Patients with a history of bladder TCC represent a distinct cohort at increased risk of developing upper tract recurrence. Prior investigation has demonstrated that prognosis for patients who develop upper tract TCC after cystectomy is poor, with these individuals typically manifesting advanced disease.[28] Studies from our institution demonstrated that in patients with a prior history of bladder cancer, cancer-specific survival was low at 71% and local recurrence-free survival was only 29%.[29] Renal preservation was still reasonable at 69%, albeit less than quoted rates of 80% in the literature.[20,25] These observations may be secondary to a distinct pathophysiologic process in a cohort with panurothelial disease. Despite these findings we feel that patients with a history of bladder TCC are a high-risk cohort with the potential of developing bilateral disease and therefore all reasonable attempts should be made to maximally preserve renal function.

One benefit of conservative therapy is that complication rates from ureteroscopic management are fairly low. Of treated individuals, 1-4% has ureteral perforation secondary to technical errors from guide wires, baskets, ureteroscopes, and/or laser fibers. Stricture rates vary from 5 to 25% in the published series[17,19,20] and are becoming less common. This decrease in stricture rates is likely due to improved technology which allows for better visualization and the employment of smaller caliber ureteroscopes. It is noteworthy, however, that not all strictures are secondary to technical error and the concern for recurrent malignancy should be excluded. Up to 40% of strictures developing after endoscopic management of upper tract TCC may represent malignancy and should therefore be biopsied.[30]

## PERCUTANEOUS MANAGEMENT

The percutaneous approach to managing upper tract TCC is generally reserved for large renal and/or proximal ureteral tumors. Advantages of this approach include the utilization of larger instruments to enhance visualization, percutaneous access to facilitate adjuvant topical therapy, improved staging capabilities, and the ability to perform second-look nephroscopy. It avoids the limitations of flexible ureteroscopy and can reliably gain access to areas that are difficult to reach, such as the lower pole calyx or the upper urinary tract in patients who have undergone prior urinary diversion. Disadvantages include the increased morbidity compared with ureteroscopy and the theoretical risk for malignant seeding of the nephrostomy tract.

Percutaneous access is obtained into the desired calyx and is described elsewhere.[31] Approach is dictated upon the location of the upper tract tumor. Tumors in peripheral calyces are best approached by establishment of a tract in direct line with the tumor. Tumors in the renal pelvis or proximal ureter may be approached with a tract in a middle or upper pole calyx to allow negotiation of the ureteropelvic junction with the nephroscope. Resection is carried out in a similar fashion to that of the ureteroscopic approach. This may be performed with biopsy forceps, cutting loop electrode or with the Nd:YAG or Ho:YAG laser fibers. A nephrostomy tube is left in place and typically a second-look nephroscopy is performed a few days later after allowing for adequate healing. The tumor base is inspected and any residual tumor is resected. If no residual disease is identified, the base should be biopsied and ablated with either electrocautery or laser fiber. The nephrostomy tube may be left in place if the patient is to undergo adjuvant topical therapy or subsequently removed several days later.

Complications from percutaneous management are more common than what is experienced from ureteroscopic approach and are similar to those experienced with treatment of benign renal disease. The most common complication is bleeding requiring transfusion and has been reported to be up to 50% in some series.[32] This was directly correlated to tumor grade since higher grade and stage tumors require deeper resection and have a higher risk for postoperative bleeding. Other complications are less common and include collecting system perforation, ureteropelvic junction obstruction, hemothorax/hydrothorax, renal failure, and malignant seeding of the nephrostomy tract. Seeding of the percutaneous tract is rare although has been reported.[33,34]

Outcomes of individuals treated with percutaneous resection of upper tract TCC strongly correlate with tumor grade. Jabbour and associates demonstrated a cancer-specific survival of 100, 94, and 63% for grades I, II, and III, respectively, in 54 patients treated percutaneously[35] and is comparable to results published in other series.[32,36] Local recurrence-free survival follows a similar pattern with respect to tumor grade. Recurrence rates for grades I, II, and III are 18, 33, and 50%.[32] When comparing outcomes of percutaneous resection to standard nephroureterectomy, Lee and associates showed no statistically significant difference in either treatment arm for overall survival.[26] Again, the most important prognostic indicator was tumor grade.

## ADJUVANT THERAPY

Adjuvant topical therapy has been investigated for treatment of upper tract TCC, similar to that performed with superficial bladder cancer. The most commonly used agents are mitomycin, thiotepa, and BCG. These agents are generally well tolerated and may be delivered percutaneously or in a retrograde fashion with a ureteral catheter following ureteroscopic resection.[37] Adjuvant BCG must be delayed for several weeks to allow for adequate healing of the urothelium to avoid BCG toxicity. The disadvantage of retrograde instillation is that cystoscopy is required at the time of each instillation. On the contrary, percutaneous access facilitates topical adjuvant therapy and minimizes contact interference between the agent and the retrograde stent yet may predispose to theoretical nephrostomy tract seeding. Nevertheless, there is no available data to advocate one approach over another. Studies have demonstrated evidence of reduced recurrence rates with administration of topical BCG.[37,38] In addition, in the only study to investigate the outcomes of individuals who received post-resection BCG and those who did not, a significantly lower recurrence rate was identified in grade I patients in the treatment arm.[35] This improvement, however, was not seen in patients with grade II or III disease. The data are promising, but no statistical improvement has yet been demonstrated with regards to recurrence or overall survival. This finding is likely secondary to the lack of individuals to provide sufficient study power and the possibility of distinct tumor biology in upper tract TCC.

## SURVEILLANCE

The potential for frequent recurrences along with grade and stage migration underscores the need for strict postoperative surveillance for upper tract TCC. Patients undergoing endoscopic resection should be followed every 3 months for the first year after treatment.[39] Chen and associates demonstrated that urine cytology and retrograde pyelography alone were insufficient in yielding adequate sensitivity for detecting upper tract recurrences and advocated the use of ureteroscopic evaluation.[40] At our institution, we now recommend every 3 month ureteroscopy for 2 years, then every 6 months for 2 years, and then yearly thereafter. Surveillance should include urine cytology, cystoscopy, ureteroscopy, and upper tract imaging during this timeframe. The contralateral upper tract should also be surveilled annually with retrograde pyelography or intravenous pyelography (IVP). Recurrent tumors are amenable to repeat endoscopic resection, but any evidence of high-grade disease or muscle invasion should be strongly considered for nephroureterectomy.

## CONCLUSION

Upper tract TCC is an uncommon malignancy that poses a significant challenge to the practicing urologist. Symptoms at presentation are variable and some individuals will manifest hematuria or flank pain, although many are asymptomatic. The yield of diagnostic modalities continues to improve but may occasionally miss the diagnosis in certain circumstances secondary to the size of the lesion or the sensitivity of the study. Currently, CT urogram is the modality of choice for imaging the upper tract, but ureteroscopic evaluation is necessary for any suspicious lesions with subsequent biopsy.

The gold-standard of therapy for upper tract TCC is nephroureterectomy with excision of a bladder cuff. However, other therapies exist and endoscopic resection has proven to be a safe and effective alternative in selective patients. These procedures are generally well tolerated and are associated with a fairly low risk for complications. Studies have demonstrated that endoscopic approach does not adversely affect overall patient survival. However, recurrences are common and may occur locally or in the bladder. Attempts to reduce recurrence risk with topical adjuvant therapy have been attempted with mitomycin, thiotepa, and BCG, but have not demonstrated a statistically significant reduction in recurrence rates. In addition to recurrence, the potential for tumor grade and stage migration are present. These findings underscore the need for strict tumor surveillance following resection and require an individual who is motivated and compliant with follow-up.
